# ALK+ Anaplastic large cell lymphoma with extensive cardiac involvement: A rare case report and review of the literature

**DOI:** 10.4322/acr.2020.231

**Published:** 2021-01-20

**Authors:** Kaniyappan Nambiyar, Kirti Gupta, Uma Debi, Saroj Kant Sinha, Rakesh Kochhar

**Affiliations:** 1 Postgraduate Institute of Medical Education and Research, Department of Histopathology, Chandigarh, India; 2 Postgraduate Institute of Medical Education and Research, Department of Radiodiagnosis and imaging, Chandigarh, India; 3 Postgraduate Institute of Medical Education and Research, Department of Gastroenterology, Chandigarh, India

**Keywords:** Lymphoma, Large-cell, Anaplastic, Anaplastic lymphoma kinase, Heart, Thymus Gland, Thromboembolism

## Abstract

Cardiac lymphoma is a rare entity. In this setting, the secondary involvement of the heart is far more frequent than the primary cardiac lymphoma. Herein, we present an autopsy case of a disseminated anaplastic lymphoma kinase (ALK)-positive anaplastic large cell lymphoma with a dominant mediastinal involvement. Extensive cardiac infiltration with the near replacement of the myocardial wall by the neoplastic cells was observed. A total of nine isolated case reports of anaplastic large cell lymphoma with cardiac involvement were found in the English-language literature, and a widespread cardiac and thymic infiltration by the systemic ALK-positive anaplastic large cell lymphoma has not been documented. An incidental regenerative nodule was also identified in the liver. The patient died of pulmonary thromboembolism and cardiac arrest.

## INTRODUCTION

Anaplastic large cell lymphoma (ALCL) is a systemic non-Hodgkin lymphoma of T-cell or null lineage, characterized by proliferation of pleomorphic cells with strongly and uniformly CD30 positivity, with membranous and Golgi pattern, initially described by Stein et al.^1^ It includes two subtypes based on the presence or absence of anaplastic lymphoma kinase (ALK) gene rearrangement on chromosome 2. The immunohistochemical expression of ALK protein and its pattern correlates with underlying genetic alterations. The ALK-positive lymphomas are more common in younger patients and carry a good prognosis, in contrast to the lymphomas, which lacks ALK expression. The assumed cell of origin of the neoplastic cell is the peripheral T-cell.^2^ Extranodal involvement may be primary or secondary as a part of systemic dissemination, the former being rarer. The usual extranodal sites are the central nervous system, gastrointestinal tract, bone marrow, skin, soft tissues, lung and liver.^3^ ^,^ ^4^ Involvement of the heart and thymus has been earlier reported in isolated case reports.^5^ ^,^ ^6^ An extensive literature search was performed using PubMed (including MEDLINE) and google scholar, and the keywords “Anaplastic large cell lymphoma,” “Anaplastic lymphoma kinase,” “Ki-1 lymphoma,” “Mediastinum,” “Heart,” “ALCL,” “Cardiac Lymphoma,” and “Thymus”. A total of nine isolated case reports of ALCL with cardiac involvement were found in the literature. This is the first autopsy case of disseminated ALK-positive ALCL with remarkable involvement of rare extranodal sites like heart and thymus.

## CASE REPORT

A 26-year-old woman presented with fever, night sweats, and shortness of breath for five days. She was initially evaluated in a private hospital and found to have massive pericardial effusion with tamponade on 2D ECHO. Pericardiocentesis was done, and one liter of straw-colored fluid was drained. The fluid analysis was suggestive of lymphocyte-predominant exudative fluid with adenosine deaminase of 33 U/L (reference range [RR]; <40 U/L). The patient was started on anti-tuberculous therapy and referred to our hospital. Her hemoglobin was 11g/dL (RR; 12.1 to 15 g/dL), with neutrophilic leukocytosis (absolute neutrophil count – 13800/µL [RR; 2000 to 7000/µL]) and platelets (156,000/µL [RR; 150,000 to 400,000/µL]). Serological tests for HBsAg, Anti HCV, and HIV were negative. A repeat 2D ECHO was suggestive of minimal pericardial effusion. A thoracic and abdominal contrast-enhanced computed tomography showed multiple enlarged cervical, axillary, and mediastinal lymph nodes, some of them showing necrosis, minimal pericardial effusion, bilateral pleural effusion, and mild ascites along with duodenal and cecal wall thickening. These findings were consistent with disseminated tuberculosis and lymphoma. Further invasive diagnostic tests could not be performed, as the patient denied consent. On the same day, the patient developed altered sensorium and breathlessness, and succumbed to her illness after five days of hospital stay, before a lymph node biopsy could be performed to establish the diagnosis.

## AUTOPSY PRESENTATION

**Figure 1:**
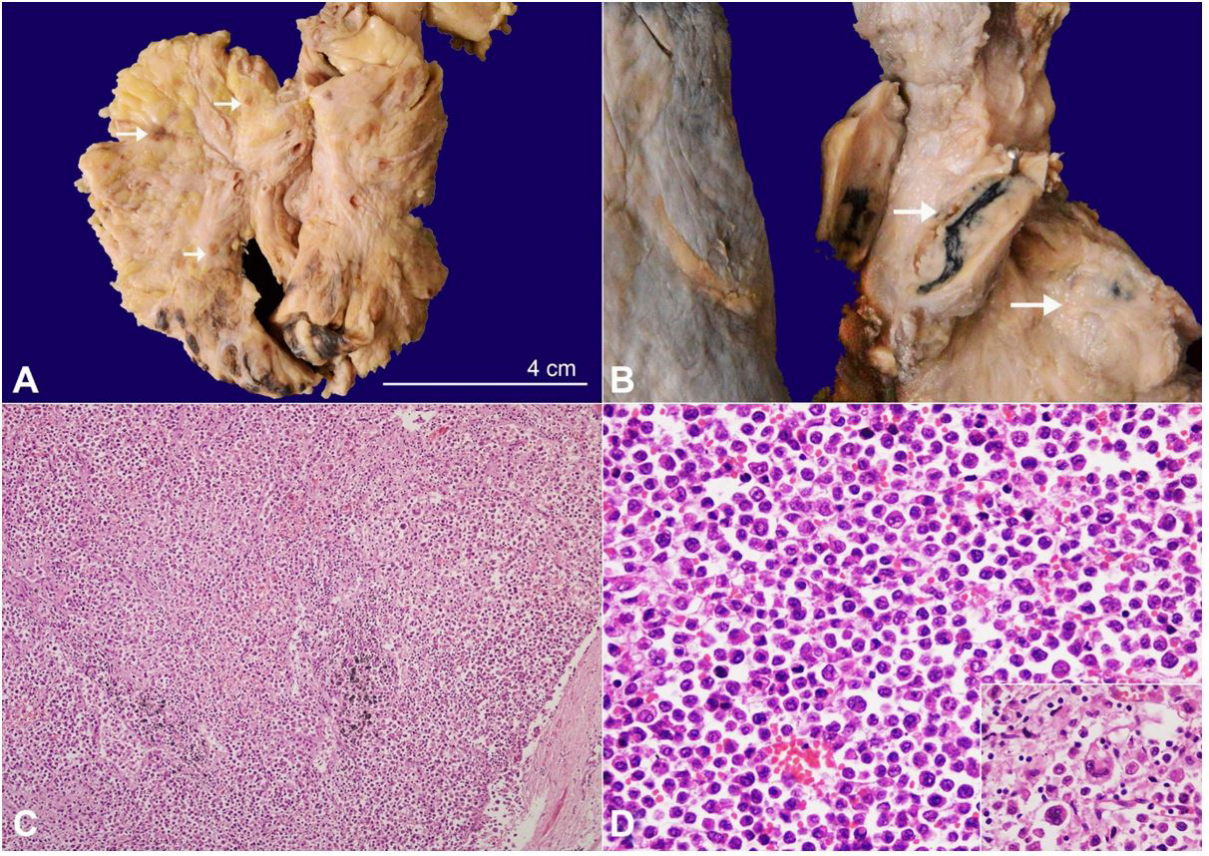
**A –** Gross view of the mesentery showing multiple enlarged lymph nodes (arrow); **B –** Gross view of the enlarged mediastinal and hilar lymph nodes (arrow). The cut surface of the lymph node is solid, and tan-yellow; **C and D –** Photomicrographs of a lymph node ; **C–** showing diffuse and sinusoidal pattern of infiltration by neoplastic lymphoid cells (H&E, 40X); D -The tumor cells are large round with abundant eosinophilic cytoplasm and enlarged hyperchromatic to vesicular nuclei. The inset shows characteristic hallmark cells with horseshoe and multilobed nuclei (H&E, 200X).

**Figure 2:**
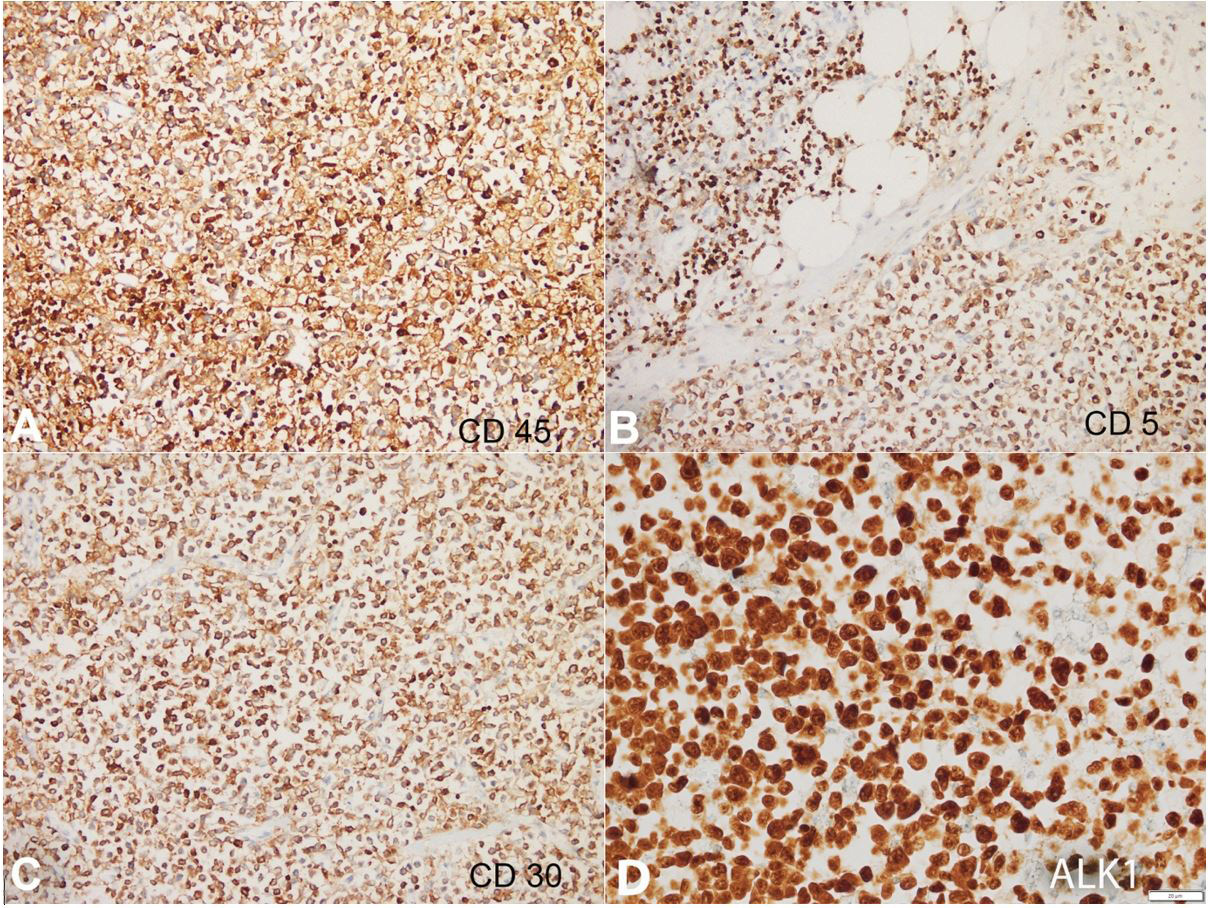
Photomicrographs of the lymph node. The tumor cells show diffuse expression of CD45 (A, 200X), CD5 (B, 200X), CD30 (C, 200X) and ALK1 (nucleocytoplasmic, D) (400X).

An autopsy was performed after informed consent. There was evidence of effusion within the pericardial, pleural, and peritoneal cavities yielding 200ml, 500ml and 1.5L of straw-colored fluid, respectively. Generalized lymphadenopathy involving mesenteric (Figure 1A), retroperitoneal, mediastinal (Figure 1B), cervical, axillary, and inguinal lymph nodes was found measuring up to 2x1x1 cm. Microscopically lymph nodes revealed diffuse infiltration by large atypical lymphoid cells along with hallmark cells having eccentric horseshoe-shaped nuclei (Figure 11D) expressing CD45, CD5, CD30, and ALK1 (nucleocytoplasmic) (Figure 2). The neoplastic cells lacked expression of CD3, CD2, CD7, TdT, CD20, and PAX5. An exceptional anterior mediastinal mass was found, which extensively infiltrated the myocardium and pericardium. The parietal and visceral pericardium were firmly adherent focally and showed solid white nodules on gross examination.

**Figure 3:**
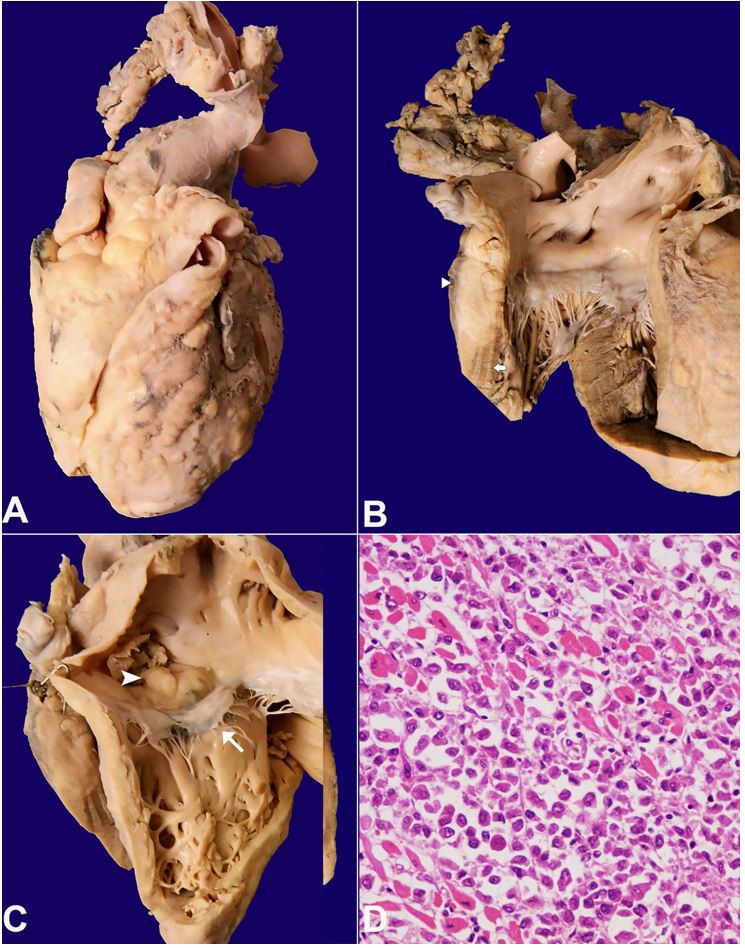
Gross view of the heart in **A–** Anterior view with multiple solid firm nodular tumor deposits involving both the ventricles and atria; **B–** View of left ventricular inflow tract showing a segment of the lateral wall with the near-complete replacement of normal myocardium (arrow) by the tumor (arrowhead); **C–** View of right ventricular inflow tract showing multiple nodular tumor infiltration involving right atrium and ventricle. A polypoidal mass of tumor (arrowhead) was seen in the right atrial cavity above the septal leaflet of the tricuspid valve (arrow); **D –** Microscopic image shows predominantly tumor cells infiltrating the cardiac myocytes (H&E, 200X).

The heart weighed 300 g (mean RR; 270 g), and showed numerous solid, firm, white nodules on the most of the visceral pericardium surface of the anterior face of the heart, with infiltration of the myocardium of both atrial and ventricular walls (Figure 3A-and 3B). A polypoidal mass of 1x1x1cm was also noted at the endocardial surface of the right atrium (Figure 3C). The microscopic examination showed diffuse infiltration of pericardium, myocardium, and endocardium by similar lymphomatous cells (Figure 3D).

**Figure 4:**
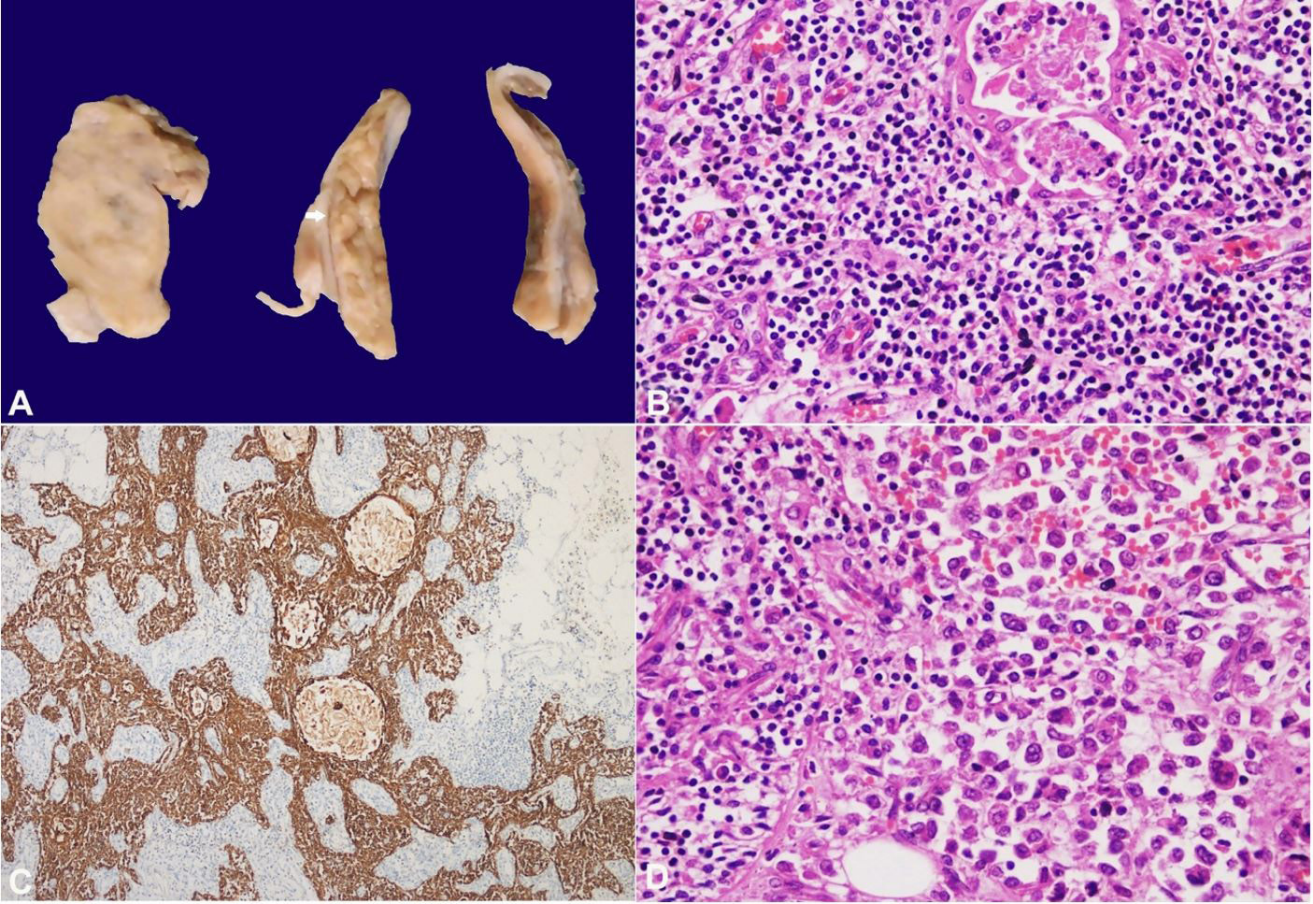
**A–** Gross image of the cut surface of the anterior mediastinal mass showing solid, tan-brown tumor (left) and lobulated normal-looking thymus (arrow); **B–** Microscopic image from normal-looking areas showing Hassall’s corpuscles and small lymphocytes (H&E, 200X); **C–** PanCK immunostain highlighting the epithelial network of the thymus (IHC, 100X); **D–** Infiltration of the thymic parenchyma (left) by the tumor cells (right) (H&E, 200X).

The sinoatrial and atrioventricular nodes were not infiltrated by the neoplastic cells. The mediastinal mass measured 5x4x1cm (Figure 4A) and microscopically revealed thymic parenchyma (Figure 4BC) with infiltration by the neoplastic cells (Figure 4D).

**Figure 5:**
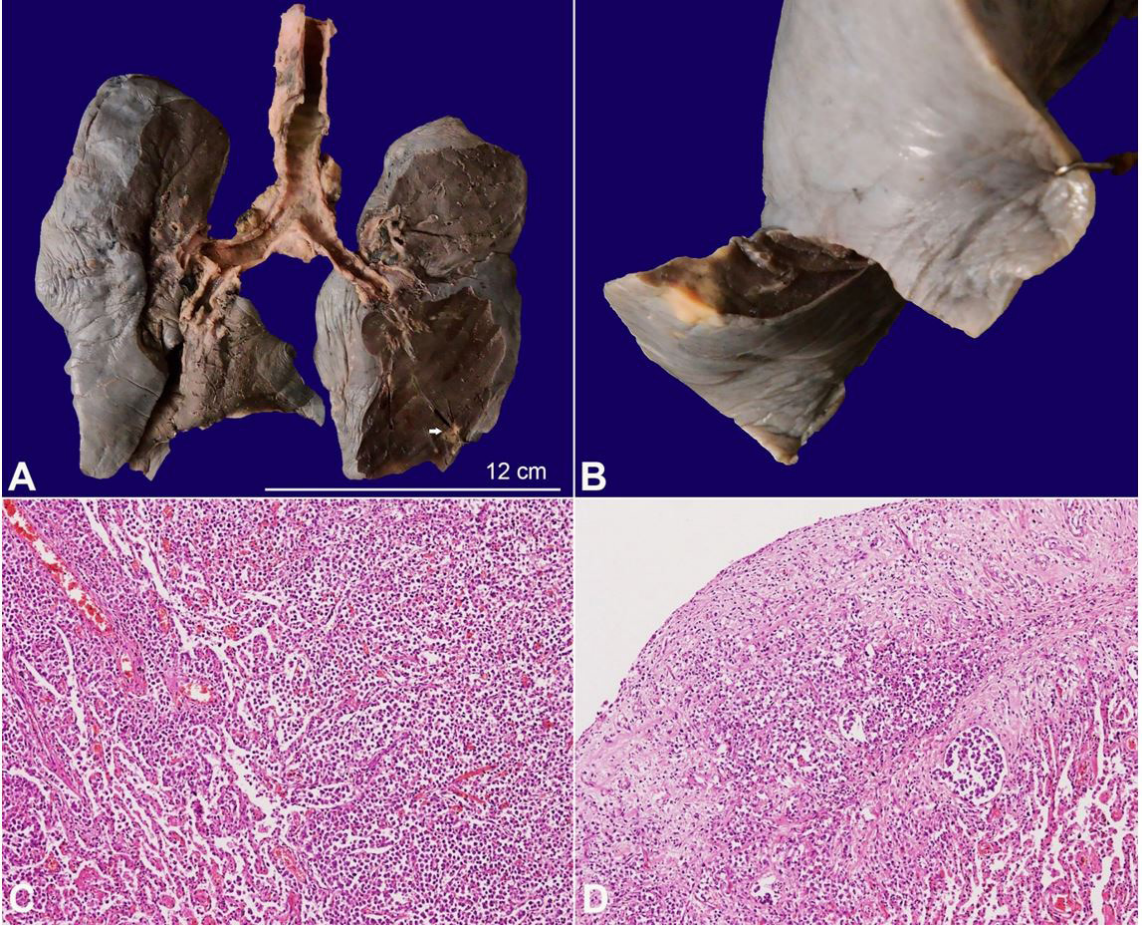
**A–** Gross image of the lungs showing pleural dullness and patchy consolidated areas. A single tan-yellow tumor deposit is also noted in the lower lobe (arrow); **B–** Closer view of a solid, yellowish subpleural tumor deposit; **C–** Microscopic image showing infiltration of the tumor cells in the alveolar cavity as well as the interstitium (H&E, 100X); **D–** The overlying visceral pleura is also infiltrated by the neoplastic cells (H&E, 100X).

Both lungs weighed 700 g (mean RR; 825 g). The pleura was dull and showed nodules in the right lower lobe (Figure 5A). The cut surfaces of the lungs were subcrepitant with few other scattered solid white nodules (Figure 5B). The major branches of the pulmonary artery, in the hilum, were occluded by fibrin thrombi. The microscopy revealed focal infiltration by the lymphoma cells involving the alveolar parenchyma and pleura (Figure 55D). Patchy areas of pulmonary edema were also noted.

**Figure 6:**
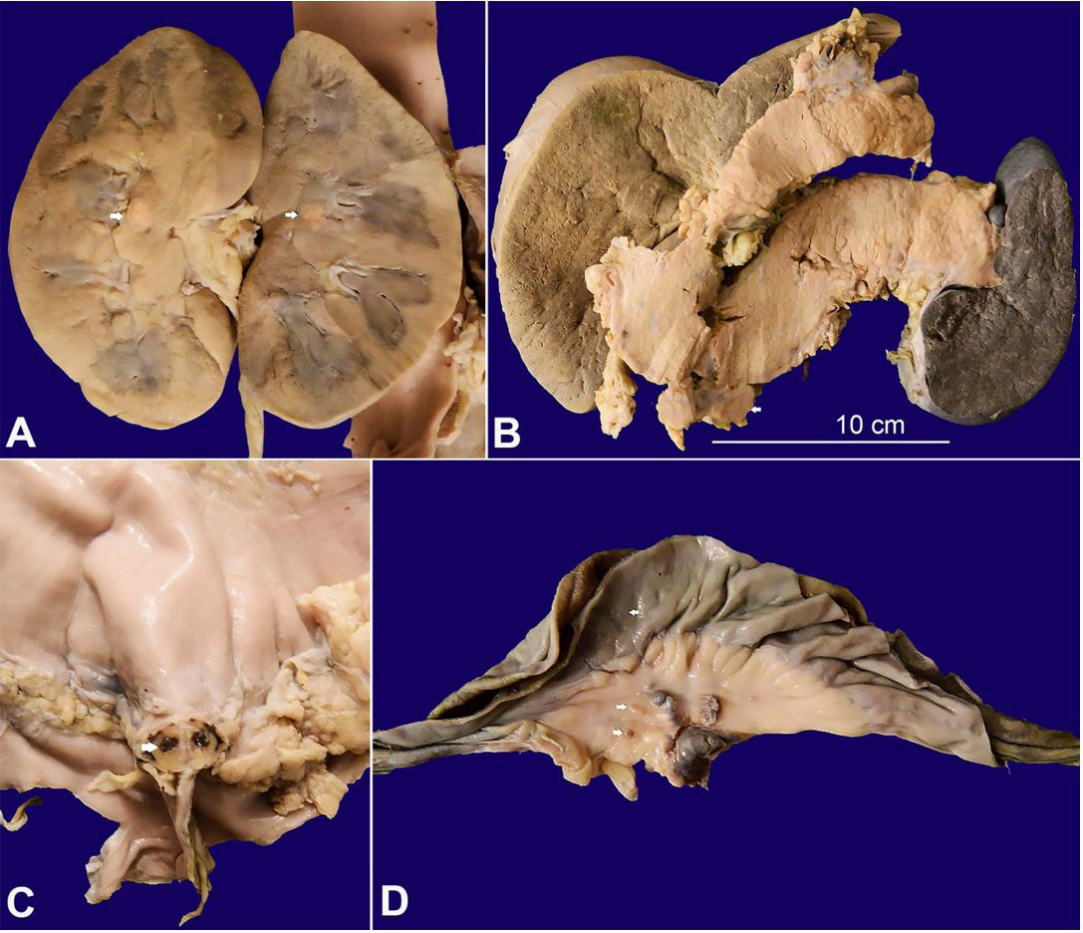
Gross view of: **A–** single yellowish tumor nodule in the medulla of the kidney (arrows); **B–** The pancreas is bulky and the peripancreatic lymph nodes are enlarged (arrows); **C–** The serosal surface of the stomach is irregular and shows yellowish-brown tumor deposit (arrow); **D–** The visceral peritoneum of small intestine and mesentery show tiny tumor deposits (arrows)

The kidney (Figure 6A), pancreas (Figure 6B), gastrointestinal tract (Figure 66D), visceral peritoneum of the ovary, retroperitoneal fat around the adrenal gland, and thyroid also showed infiltration by the tumor.

**Figure 7:**
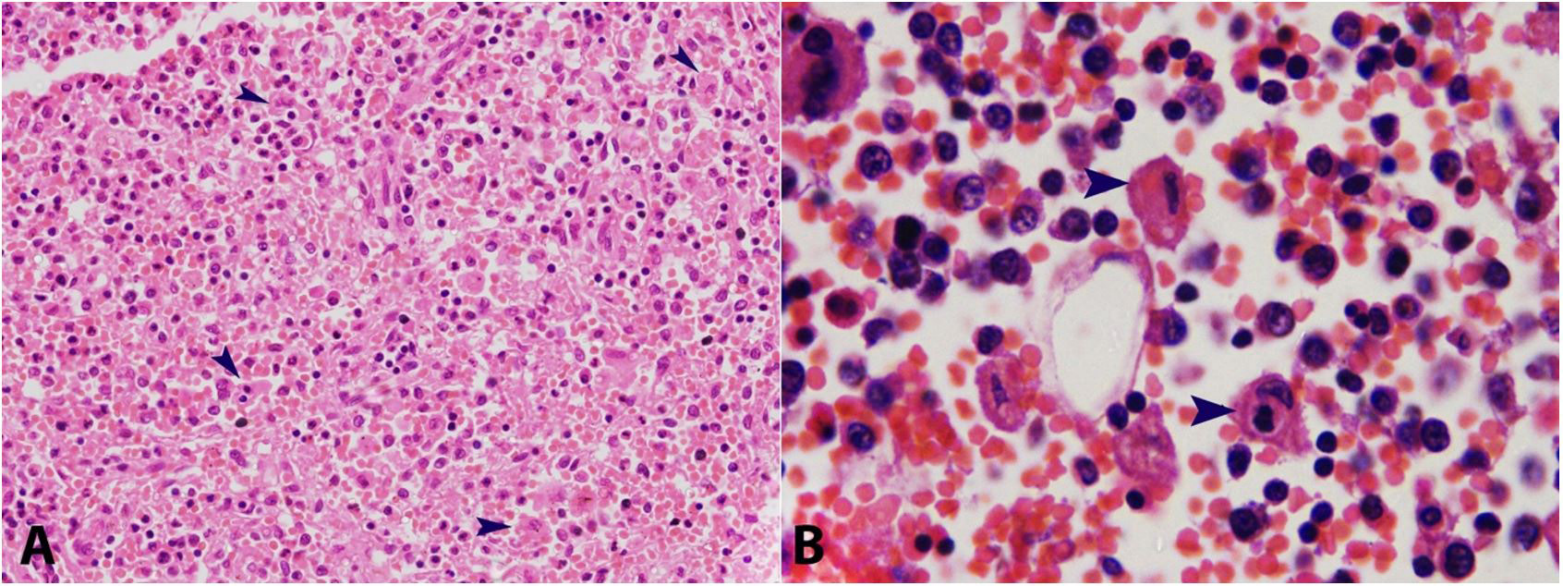
**A–** Photomicrograph of the spleen showing increased histiocytes and hemophagocytosis (arrowhead) (H&E, 200X); **B–** Photomicrograph of the bone marrow showing hemophagocytosis (arrowhead) (H&E, 1000X).

There was intravascular lymphomatosis (blood and lymphatic vessels), along with perineural infiltration. In addition, kidneys showed ischemic acute tubular necrosis. The liver showed nutmeg appearance and an incidental solitary nodule, which was microscopically found to be a regenerative nodule. The spleen was grossly unremarkable. Histiocytosis with secondary hemophagocytosis was noted in the spleen, lymph nodes, and bone marrow (Figure 7). There was no morphological and immunohistochemical evidence of infiltration by neoplastic cells in the liver, spleen, and bone marrow. The terminal event that led to death was major pulmonary arterial thromboembolism and shock. No focus of active or chronic tuberculosis was noted.

## CLINICAL DISCUSSION

ALCL accounts for 3% of the non-Hodgkin lymphoma occurring mostly in adults, and

10-20% occur in Pediatrics.^5^ In adults, the mediastinal involvement of ALCL occurs in 7%off the cases,^7^ while in Pediatrics, this site is involved in about half of the cases.^8^ Cardiac lymphoma is also rare. However, the secondary involvement has an incidence that ranges between 10 to 30% among all types of lymphoma.^6^ Primary cardiac lymphomas have an incidence of less than 1% and are diagnosed only in the absence of nodal disease with initial cardiac involvement.^6^ The characteristics of cardiac ALCLs reported in literature^3^ ^-^ ^6^ ^,^ ^9^ ^-^ ^13^ are enumerated in Table 1.

**Table 1:** Review of ALCL cases with cardiac involvement

**Authors**	**Age/ sex**	**Nature of disease**	**Cardiac involvement**	**Extracardiac involvement**	**Positive reactions**
**Muthusamy** **^3^**	22/M	Secondary	Pericardium	Cervical lymph node	CD30, EMA and ALK
**Lim** **^4^**	29/M	NA	Pericardium, both ventricles	Skin, lung and mediastinum	CD30 and ALK
**Nagai** **^5^**	11/M	Secondary	Pericardium and left ventricle	Left mandibula and 3^rd^ rib	CD3, Granzyme B, CD56, CD30 and ALK
**Punnoose** **^6^**	21/M	Secondary	Pericardium and myocardium	Generalized lymphadenopathy	CD30, ALK, clusterin, endomysial antibodies, CD 43, CD4, CD7, granzyme, perforin, and TIA-1
**Papadopoulou** **^9^**	6/M	NA	Endocardium	Thymus	CD30 (ALK status not mentioned)
**Lauten** **^10^**	8/M	Primary	Both ventricles	Absent	CD30, CD2, CD3, granzyme B, perforin, EMA and ALK
**Mira-Perceval** **^11^**	2/M	Secondary	Pericardium	Skin and lymph node	CD30 and ALK
**Narayanan** **^12^**	14/F	Secondary	Anterior mediastinum	Cervical lymph node, Sternum	CD30, EMA and ALK
**Rannan-Eliya** **^13^**	14/F	Secondary	Right atrium and ventricles	Skin without nodal involvement	ALK (CD30 status not mentioned)
**Index case**	26/F	Secondary	Pericardium, atria and ventricles	Lymph nodes, thymus, lungs, Pancreas, kidney, gastrointestinal tract, thyroid and visceral peritoneum of ovary	CD45, CD5, CD30 and ALK

ALK, Anaplastic lymphoma kinase; EMA, Epithelial membrane antigen; F= female; IHC= immunohistochemistry; M=male; NA= non-available; TIA= T-cell intracytoplasmic antigen

The present case was categorized as secondary cardiac lymphoma with dominant mediastinal involvement. Secondary cardiac involvement results from either retrograde lymphatic extension, hematogenous, or direct contiguous extension.^14^ The involvement of other organs, and myocardial infiltration suggests the hematogenous spread. Thymic involvement by ALCL is unusual, with only a single case report in the literature.^9^ However, we presume this may not represent the true incidence of the thymic involvement, as most of the patients are treated successfully, and mediastinal biopsy is not performed for the diagnosis. Interestingly, a few recent studies argue against the presently accepted origin of ALK-positive ALCL from peripheral mature T-cells and propose a thymic origin with a peripheral presentation.^2^ ^,^ ^15^ The thymic involvement in the index case may be related to the proposed thymic origin.

Patients with secondary cardiac involvement usually present with cardiac symptoms like progressive heart failure, arrhythmia, chest pain, and syncope, based on the exact location of the tumor.^5^ However, despite extensive cardiac involvement, cardiac symptoms were not the initial presentation of the index case. The hemophagocytosis associated with ALCL, seen in this patient, is exceedingly rare, and if present, portends a poor prognosis.^16^ Interestingly, there was no infiltration of the liver, spleen, or bone marrow by the neoplastic cells. Another unusual finding observed was an incidental regenerative nodule in the liver.

About 15-85% off the nodal ALCLs are associated with nucleophosmin *(NPM)1*-*ALK* gene fusion, which can be detected by using ALK-1 immunostain.^17^ ALK protein expression is seen in most of the pediatric ALCLs.^5^ Among the ALK-positive ALCL, 15% of the cases show chromosomal rearrangement involving *ALK* gene with different partners other than *NPM1* gene.^17^ Nucleocytoplasmic immunostaining, seen in this case with ALK1, indicates the ALK fusion partner to be NPM1. CD3 signaling cascade is downregulated in an *NPM1–ALK*-dependent manner in ALK-positive ALCL,^15^ as observed in our case with the lack of CD3 expression. The T-cell origin of the neoplasm in the present case was established with the positive expression of CD5.

Although ALK-positive ALCLs have a favorable prognosis,^5^ presence of predominant extranodal involvement, intravascular lymphomatosis, and hemophagocytosis are associated with poor outcome.^16^ Death in secondary cardiac neoplasms may be due to cardiac tamponade, congestive heart failure, coronary artery invasion, or sinoatrial node invasion.^14^ However, the death in the index case was associated with thromboembolism in the major pulmonary artery. Incidence rates of thrombosis in patients with lymphoma vary between 2% and 13%.^18^ Studies by Hohaus et al.^19^ and Santi et al.,^20^ including a large number of Non-Hodgkin lymphomas, revealed that ALCL was never reported to have venous thromboembolism. Arterial thromboembolism has been reported in an ALK-positive ALCL with cardiac involvement during chemotherapy by Nagai et al.^5^

## CONCLUSION

The present case is an uncommon case of disseminated ALK-positive ALCL in a young adult with extensive cardiac and thymic infiltration. While the cardiac presentation has been described as case report in the literature, such a dominant cardiac infiltration accompanied by thymic involvement and pulmonary thromboembolism has not been reported so far.
